# Obesity-related knowledge, attitude, and practices among primary care physicians in China: a cross-sectional study

**DOI:** 10.3389/fmed.2025.1668886

**Published:** 2025-09-25

**Authors:** Jusumei Tang, Haoyuan Chen, Linhua Pi

**Affiliations:** ^1^Department of Endocrinology and Metabolism, The Second Affiliated Hospital of Guilin Medical University, Guilin, Guangxi, China; ^2^Guangxi Clinical Research Center for Diabetes and Metabolic Diseases, The Second Affiliated Hospital of Guilin Medical University, Guilin, Guangxi, China; ^3^Guangxi Health Commission Key Laboratory of Glucose and Lipid Metabolism Disorders, The Second Affiliated Hospital of Guilin Medical University, Guilin, Guangxi, China

**Keywords:** obesity, knowledge, attitude, practice, primary care providers, Southwest China, cross-sectional study

## Abstract

**Background:**

Obesity is a major global public health crisis but remains inadequately addressed during clinical encounters. In China, the primary healthcare system plays a vital role in managing chronic; however, the knowledge, attitude, and practice (KAP) of primary care physicians (PCPs) regarding obesity have not been previously described.

**Methods:**

This cross-sectional study surveyed 240 PCPs in Southwest China using self-administered KAP questionnaires. The questionnaires assessed obesity-related KAP and were measured using SPSS software. Descriptive statistics and univariate analyses were used to analyze the data.

**Results:**

In total, 240 PCPs completed the survey. While the majority (75.0%) reported awareness of the adverse health effects of obesity, their attitudes toward obesity management were generally negative. Notably, significant gaps were found in both knowledge and practice. Obesity knowledge of PCPs and practice subscale scores were 45.9% and 23.1% of the corresponding optimal scores, respectively. Exposure to formal obesity-related training was associated with significantly higher scores in knowledge (3.95 ± 2.04 vs. 2.32 ± 2.16, *p* < 0.001), attitudes (4.59 ± 1.12 vs. 3.96 ± 1.18, *p* < 0.001), and practices (1.76 ± 1.06 vs. 1.25 ± 1.00, *p* = 0.001).

**Conclusion:**

Substantial gaps exist in the KAP of PCPs regarding obesity in Southwest China. Formal training appears to significantly improve obesity-related KAP and may enhance the primary care management of patients with obesity.

## Background

Obesity is a chronic disease that poses serious health risks and constitutes a major public health crisis globally (1). Consistent with global trends, obesity has become increasingly prevalent in China due to rapid economic and social changes since the 1990s. According to data from the recent national survey indicated that the prevalence of obesity and overweight were 14.1% and 34.8%, respectively among adults living in China (2).

Obesity has substantial health, economic, and social consequences. It is associated with a high risk of numerous chronic conditions, including diabetes, hypertension, dyslipidemia, cardiovascular disease, non-alcoholic fatty liver disease, and various cancers (1, 3). A high body mass index is also associated with increased premature mortality (4), with more than five million deaths globally attributed to overweight and obesity annually (5). Obesity is prevalent among special group, such as seafarers (6, 7), childhood obesity (8), etc. Specific initiatives should be taken to curb the rising tide of obesity.

In response to the escalating obesity burden, the Chinese government has made numerous efforts to raise awareness, promote healthy lifestyles, and improve dietary habits (9, 10). Despite these efforts, the prevalence of obesity among Chinese adults has not been curbed. Recently, the government of China launched the “Weight Management Year” plan as a more targeted effort to curb rising obesity rates (11). Physicians are expected to play a central role in obesity management; however, obesity often remains inadequately addressed in clinical encounters, primarily due to inadequate physician knowledge and training (12–14).

Primary healthcare (PHC) systems are pivotal in addressing chronic diseases in China (15), with most patients, including those with obesity, receive healthcare services from PHC facilities (16). Nevertheless, many primary care physicians (PCPs) in China lack obesity-related training, and obesity is often overlooked in routine care. Studies conducted in the United States have indicated lack of confidence in managing obesity among PCPs (13). Related studies in China have shown that nurses (17), rural residents (18) and university students (19) generally possess limited knowledge about obesity and its metabolic complications. A recent study conducted to identify the perceptions, attitudes, and barriers to effective obesity care among healthcare professionals in China found that while most acknowledged obesity as a chronic disease, they often refrained from discussing it with patients for lacking of confidence in managing obesity (20).

To the best of our knowledge, no studies have yet evaluated the knowledge, attitudes, and practices (KAP) related to obesity among PCPs in China. Therefore, this study aimed to assess obesity-related KAP among PCPS in Southwest China and to examine associations between provider characteristics and these outcomes.

## Methods

### Sample and survey administration

This descriptive cross-sectional study used a convenience sampling approach and was conducted among PCPs affiliated with 14 township health centers in Guilin City, Guangxi Zhuang Autonomous Region, from February 12 to May 20, 2025. Board-certified PCPs actively providing primary care services were included. Those holding solely administrative roles or working in support departments, such as pharmacies and laboratory services, were excluded.

Using the OpenEpi web-based calculator, a minimum sample size of 195 PCPs was estimated, assuming a margin of error of 0.05 and 95% confidence interval, and a population size of 800. Considering 10% contingency, the minimum required sample size was 216. A simple random sampling method was used to select 500 PCPs from the 14 health centers.

### Survey instrument

A structured questionnaire was developed to assess the KAP of participants regarding obesity, based on previous literature with minor modifications (21, 22). The questionnaire consisted of five sections. The first collected demographic characteristics, including sex, age, education level, professional title, years of clinical experience, and prior participation in obesity-related training programs. The second section assessed knowledge of obesity using six main questions, such as those addressing the pathophysiological mechanisms of obesity. The third section evaluated attitudes toward obesity, while the fourth section evaluated the clinical practices of PCPs related to obesity management.

Responses were recording using a 4-point Likert scale tailored to each domain (1 = high/moderate, strongly agree/agree, frequently/sometimes; 0 = low/very low, strongly disagree/disagree, rarely/never). Two experts in endocrinology evaluated the questionnaire items for difficulty and clarity and five lay participants reviewed the questionnaire to confirm that it accurately covered all aspects of the topic. Reliability was assessed using Cronbach's alpha, which demonstrated acceptable internal consistency, confirming that the questionnaire's scales consistently measured the intended constructs. A pre-test was conducted among 20 PCPs to refine the questionnaire for reliability, clarity, and interpretability. The instrument demonstrated good internal consistency, with an overall Cronbach's alpha value of 0.835 (0.825, 0.867, and 0.842 for knowledge, attitudes, and practices, respectively).

### Ethical approval

This study was conducted in accordance with the Declaration of Helsinki and received ethical approval from The Second Affiliated Hospital of Guilin Medical University. An informed consent statement was included at the beginning of the online questionnaire, outlining the voluntary nature of participation and the confidentiality of responses. All participants provided informed consent.

### Statistical analysis

Data were extracted and analyzed using SPSS version 25.0 (IBM Corporation, Armonk, NY). Descriptive data were expressed as numbers, percentages, means, and standard deviations, as appropriate. Associations between PCP characteristics and KAP scores were evaluated using chi-square tests and t-tests, as appropriate. Statistical significance was set at *p* < 0.05.

## Results

### General characteristics of the study sample

Of the 500 PCPs invited, 240 voluntarily participated and returned completed questionnaires, yielding a response rate of 48.0%. The characteristics of the study participants are summarized in Table 1. The mean age was 38.6 ± 9.3 years, with nearly half of the participants aged ≥40 years (*n* = 114, 47.5%). Most participants were females (*n* = 174, 65.5%). The PCPs included 137 residents, 63 attending physicians, and 40 senior physicians. Those with more than 20 years of clinical experience represented the largest subgroup (*n* = 86, 35.9%). Only 26.2% of respondents reported having received formal obesity-related training.

**Table 1:** Demographic and characteristics of participants (*N* = 240).

**Provider characteristics**	**Mean ±SD**	**Number (*n*)**	**Percentage (%)**
**Sex**			
Male		66	27.5
Female		174	72.5
Age (years)	38.6 ± 9.3		
<40		126	52.5
≥40		114	47.5
**Professional titles**			
Resident physicians		137	57.1
Attending physicians		63	26.2
Senior physicians		40	16.7
Duration of practice (years)	15.5 ± 9.7		
<10 years		69	28.8
10–20 years		85	35.5
≥20 years		86	35.9
**Training on obesity management**			
No		177	73.8
Yes		63	26.2

SD, standard deviation.

### Self-reported knowledge of PCPs regarding obesity

As shown in Table 2, nearly half of the participants reported having a high or moderate level of knowledge regarding obesity pathophysiology (48.7%), causes of obesity (56.2%), dietary and nutritional recommendations for obese patients (55.0%), and behavioral strategies for weight management (59.5%). However, knowledge was lower concerning indications for bariatric surgery and anti-obesity medications, with only 25.8% and 30.0% reporting high or moderate knowledge levels, respectively.

**Table 2:** Knowledge of PCPs regarding obesity (*N* = 240).

**Item**	**High/moderate level (%)**	**Score (0–1)mean ±SD**
K1. Obesity pathophysiology	117 (48.7)	0.49 ± 0.50
K2. Causes of obesity	135 (56.2)	0.56 ± 0.50
K3. Diet and nutrition recommendations for patients with obesity	132 (55.0)	0.55 ± 0.50
K4. Behavior changes to help with weight management	143 (59.5)	0.60 ± 0.49
K5. Indications for bariatric surgery	62(25.8)	0.26 ± 0.44
K6. Indications and usage of anti-obesity medications	72 (30.0)	0.30 ± 0.46
Total	661(45.9%)	2.75 ± 2.19

PCPs, primary care physicians; SD, standard deviation.

### Attitudes of PCPs regarding obesity

As shown in Table 3, majority of PCPs recognized obesity as a chronic disease (*n* = 192, 80.0%) that affects almost every aspect of the life of the patient (*n* = 180, 75.0%). Meanwhile, 166 (69.1%) agreed that obesity involves multifactorial causes and that obese people should not be solely blamed for their weight. The majority of participants (*n* = 200, 83.3%) considered diet and exercise as the cornerstone of obesity management. However, only 128 (53.3%) viewed obesity management as part of their routine duties, and half of the PCPs felt that discussing weight issues was likely to offend patients.

**Table 3:** Attitude of PCPs regarding obesity (*N* = 240).

**Item**	**Strongly agree or agree (%)**	**Score (0–1) mean ±SD**
A1. Obesity is a chronic disease	192 (80.0)	0.80 ± 0.40
A2.Obesity affects almost every part of a person's life.	180 (75.0)	0.75 ± 0.43
A3. Fat people are not only have themselves to blame for their weight.	166 (69.1)	0.69 ± 0.46
A4. Diet and exercise are important for obesity management	200 (83.3)	0.83 ± 0.37
A5. Obesity management is part of the scope of primary care	128 (53.3)	0.53 ± 0.49
A6. Discussing weight is unlikely to offend patients	124 (51.6)	0.52 ± 0.50
Total	982 (68.8)	4.13 ± 1.20

PCPs, primary care physicians; SD, standard deviation.

### Practice of PCPs regarding obesity

As shown in Table 4, 118 (49.1%) PCPs reported addressing obesity during daily consultations. Comprehensive counseling on diet and nutrition was provided by 30.8% of PCPs, and 29.5% offered behavioral and medication advice. In addition to lifestyle changes, only 6.2% had prescribed anti-obesity medicines, and 7.9% had referred patients for bariatric surgery. Only 34 (14.5%) participants acknowledged the psychological impact of obesity and provided relevant counseling to patients.

**Table 4:** Practice of PCPs regarding obesity (*N* = 240).

**Item**	**Frequently or sometimes (%)**	**Score (0–1) mean ±SD**
P 1. During my daily work, I have addressed and discussed obesity as a problem with my patients.	118 (49.1)	0.49 ± 0.50
P2. During my daily work, I have provided counseling on diet and nutrition for patients with obesity.	74 (30.8)	0.31 ± 0.46
P3. During my daily work, I have provided motivational interviewing for behavior changes to help with weight management for patients with obesity.	71 (29.5)	0.29 ± 0.46
P4. During my daily work, I have provided psychological counseling for patients with obesity.	35 (14.5)	0.15 ± 0.35
P5. During my daily work, I have prescribed anti-obesity medicines for patients with obesity.	15 (6.2)	0.06 ± 0.24
P6. During my daily work, I have referred to bariatric surgery for patients with obesity.	19 (7.9)	0.08 ± 0.27
Total	332 (23.1)	1.38 ± 1.04

PCPs, primary care physicians; SD, standard deviation.

### Factors associated with the overall KAP scores

As shown in Table 5, significant associations were found between professional title and formal obesity training with knowledge scores. Resident physicians had the highest knowledge scores, whereas attending physicians had the lowest. Physicians with formal obesity training scored significantly higher than those without training. Formal obesity training was also a significant predictor of better attitudes toward obesity management. In terms of practice, male gender, age <40 years, lower professional title, shorter duration of practice, and formal training were all positively correlated with better obesity-related practices among PCPs.

**Table 5:** Association between overall KAP scores and participant characteristics.

**Provider characteristics**	***n***	**Knowledge score**		**Attitude score**		**Practice score**	
		**Mean (SD)**	***p***	**Mean (SD)**	***p***	**Mean (SD)**	***p***
Sex			0.062		0.225		0.001
Male	66	3.18 ± 2.26		4.30 ± 1.17		1.72 ± 1.03	
Female	174	2.59 ± 2.15		4.05 ± 1.20		1.25 ± 1.01	
Age (years)			0.815		0.225		0.001
<40	126	2.79 ± 2.23		4.21 ± 1.10		1.60 ± 1.04	
≥40	114	2.72 ± 2.15		4.03 ± 1.29		1.14 ± 0.99	
Professional titles			0.001		0.066		<0.001
Resident physicians	137	3.16 ± 2.25		4.28 ± 1.24		1.73 ± 0.99	
Attending physicians	63	1.96 ± 1.92		3.97 ± 1.09		1.02 ± 1.01	
Senior physicians	40	2.60 ± 2.00		3.85 ± 1.17		0.78 ± 0.77	
Duration of practice (years)			0.186		0.628		0.03
<10 years	69	2.99 ± 2.17		4.21 ± 1.19		1.72 ± 0.97	
10–20 years	85	2.41 ± 2.11		4.04 ± 1.16		1.29 ± 1.06	
≥20 years	86	2.91 ± 2.26		4.14 ± 1.25		1.20 ± 1.03	
Training on obesity management			<0.001		<0.001		0.001
No	177	2.32 ± 2.04		3.96 ± 1.18		1.25 ± 1.00	
Yes	63	3.95 ± 2.16		4.59 ± 1.12		1.76 ± 1.06	

KAP, knowledge, attitudes, and practices; SD, standard deviation.

## Discussion

Obesity is a major global public health crisis (1). PCPs are the first point of contact for patients and are expected to have sufficient KAP regarding obesity management. Previous studies have shown that PCPs often find obesity management challenging and do not consistently address it during clinical encounters (14, 23, 24). This study aimed to evaluate the KAP of PCPs in China regarding obesity management. Our findings demonstrated that most physicians claimed to be aware of the harmful effects of obesity, but their attitudes usually deemed obesity management is out of the scope of primary care and discussing weight is unlikely to offend patients, possibly due to heavy workloads or a lack of competence. Notably, a substantial gap was noted in knowledge and practices. Formal training in obesity management emerged as a significant predictor of KAP.

Although obesity is recognized as a consequence of caloric surplus (25), emerging evidence highlights the role of complex, interconnected etiological drivers, such as genetic predispositions, gut dysbiosis, and obesogenic environments that disrupt metabolic homeostasis (26). Our study demonstrated that PCPs only had a moderate level of self-reported knowledge regarding the pathophysiology and cause of obesity: 48.7% and 56.2% of participants rated their understanding of obesity pathophysiology and causes, respectively, as high or moderate. Another study conducted in Sweden indicated that most physicians deemed obesity was a chronic disease (91%), obesity regulation sits in the hypothalamus (70%) and obesity was due to disorders of appetite regulation (69%), which was higher than this study, this may be attributed to difference in methodologies and variations in geographical and economic characteristics (27). Our study utilized a 4-point Likert scale, while the study conducted in Sweden used multiple-choice questions. Regarding obesity management, around half of the participants were familiar with lifestyle interventions, such as diet and behavioral modifications, aligning with prior research among healthcare professionals in China (20). However, PCPs demonstrated poor knowledge of pharmacotherapy and bariatric surgery for obesity, possibly due to the absence of national guidelines and limited training opportunities. Our findings are comparable with studies from the United States (21) and Poland (28) which also reported large gaps in obesity management. Exposure to formal obesity-related training was a significant predictor of better knowledge, consistent with previous reports (21).

Most PCPs viewed obesity as a chronic disease and acknowledged its serious impact. Furthermore, half of the PCPs agreed that obesity involves multifactorial causes and that individuals are not solely responsible for their weight. These attitudes are consistent with findings from studies in Lebanon (29) and Saudi Arabia (30). Most respondents also considered diet and exercise as fundamental components of obesity management (29–31). However, only half of the participants thought that obesity management was part of their daily work and many were concerned that discussing weight was likely to offend patients. A Swedish study showed a similar attitude, with many physicians believing that weight management was primarily the responsibility of the patient (27). In contrast, a study in Norway found that most physicians regarded obesity management as part of their daily work. These discrepancies can be attributed to variations in geographical and socioeconomic factors. As in previous research (21), our study confirmed that formal training positively influenced the attitudes of PCPs toward obesity management.

This study also revealed generally poor obesity-related practices among PCPs. Only half of the PCPs routinely discuss obesity with patients, and even fewer have provided comprehensive counseling on diet and nutrition, behavioral changes, and psychological counseling. Prescribing anti-obesity medication and referring patients for bariatric surgery were also uncommon. These deficiencies may be attributed to the following reasons: (1) the lack of time and resources during consultations and lack of obesity-related training, which has also been postulated by previous studies (13, 27); (2) possibly due to the absence of national guidelines and limited training opportunities. Interestingly, male PCPs performed better than female PCPSs. In contrast to our findings, a study conducted in the United States reported that female PCPs had superior practices toward obesity management than male (32), while another study found no sex-based differences (33). Our results also indicated that younger age, lower professional titles, and shorter duration of practice were positively associated with better practice. In the PHC system of China, these factors are often interrelated. The association between age and obesity-related practice is consistent with a study conducted in Sweden (27). Younger physicians may be more open to recent guidelines and more willing to engage in training, which could explain these associations. Exposure to formal obesity training also emerged as a strong predictor of improved practice (21).

This study has several limitations. First, it was conducted in Southwest China, and its findings may not be generalizable due to geographic and sociocultural differences. Second, the self-reported nature of the KAP survey may have introduced recall and social desirability biases, potentially leading respondents to overstate positive attitudes and practices toward obesity. Further studies addressing these limitations should be done to validate our findings.

## Conclusion

This study highlights substantial gaps in the KAP of PCPs regarding obesity management in Southwest China. The results underscore the importance of formal training to enhance the capacity of PCPs to better manage and treat patients with obesity. Future efforts aimed at improving knowledge and attitudes regarding obesity treatment among PCPs could have important public health implications.

## Data Availability

The raw data supporting the conclusions of this article will be made available by the authors, without undue reservation.
