# Supplementary material for: Obesity-related knowledge, attitude, and practices among primary care physicians in China: a cross-sectional study
Source: Front Med (Lausanne). 2025 Sep 25;12:1668886. doi: 10.3389/fmed.2025.1668886 (PMC12507768; doi:10.3389/fmed.2025.1668886)
Supplement: Supplementary file 1 [file Table_1.docx]

Obesity-related knowledge, attitude, and practices among primary care physicians in China

I. Basic Information

Age:

Gender: □ Male □ Female

Professional titles: □ Resident physicians □ Attending physicians □ Senior physicians

Years of Experience: □ Less than 10 years □ 10-20 years □ More than 20 years

Did you participate in any continuing medical education on obesity: □ Yes □ No

II.Self-reported knowledge of PCPs regarding obesity

1.Obesity pathophysiology

□ very low □ Low □ Moderate □ High

1. Causes of obesity

□ very low □ Low □ Moderate □ High

1. Diet and nutrition recommendations for patients with obesity

□ very low □ Low □ Moderate □ High

1. Behavior changes to help with weight management

□ very low □ Low □ Moderate □ High

1. Indications for bariatric surgery

□ very low □ Low □ Moderate □ High

1. Indications and usage of anti-obesity medications

□ very low □ Low □ Moderate □ High

III. Obesity attitude

1. Obesity is a chronic disease

□ strongly disagree □ Disagree □ Agree □ Strongly agree

1. Obesity affects almost every part of a person’s life.

□ strongly disagree □ Disagree □ Agree □ Strongly agree

1. Fat people are not only have themselves to blame for their weight.

□ strongly disagree □ Disagree □ Agree □ Strongly agree

1. Diet and exercise are important for obesity management

□ strongly disagree □ Disagree □ Agree □ Strongly agree

1. Obesity management is part of the scope of primary care

□ strongly disagree □ Disagree □ Agree □ Strongly agree

1. Discussing weight is unlikely to offend patients

□ strongly disagree □ Disagree □ Agree □ Strongly agree

IV. Obesity practice

1. During my daily work, I have addressed and discussed obesity as a problem with my patients.

□ Never □ Rarely □ Some □ Frequently

1. During my daily work, I have provided counseling on diet and nutrition for for patients with obesity.

□ Never □ Rarely □ Some □ Frequently

1. During my daily work, I have provided motivational interviewing for behavior changes to help with weight management for for patients with obesity.

□ Never □ Rarely □ Some □ Frequently

1. During my daily work, I have provided psychological counseling for patients with obesity.

□ Never □ Rarely □ Some □ Frequently

1. During my daily work, I have prescribed anti-obesity medicines for patients with obesity.

□ Never □ Rarely □ Some □ Frequently

1. During my daily work, I have referred to bariatric surgery for patients with obesity.

□ Never □ Rarely □ Some □ Frequently
